# Anaphylaxis to additives in vaccines

**DOI:** 10.1007/s40629-022-00215-8

**Published:** 2022-06-14

**Authors:** Vera Mahler, Ann-Christine Junker

**Affiliations:** Paul-Ehrlich-Institut, Paul-Ehrlich-Straße 51–59, 63225 Langen, Germany

**Keywords:** Excipients, Residues, Contamination, IgE, Pseudoallergy

## Abstract

Anaphylaxis in connection with the administration of vaccines occurs only very rarely. Triggers of immunoglobulin IgE-mediated and non-IgE-mediated anaphylaxis—in addition to the active ingredient itself—may be excipients contained in the vaccine due to their special properties. Some of the excipients in medicinal products are the same compounds used as additives in food. Furthermore, residues from the manufacturing process (e.g., chicken egg white, casein, antibiotics, formaldehyde) or contaminants (e.g., from the primary packaging material) may be potential triggers of anaphylaxis in vaccines. This review article provides an overview of ingredients in vaccines that pose an allergenic risk potential. The components of COVID-19 vaccines approved and marketed in Germany are discussed with regard to their potential for triggering anaphylaxis and possible pathophysiological mechanisms involved.

## Definition of “additives” and “excipients”

Additives are natural or artificial substances with or without nutritional value, which are used for the production of foods and beverages [1]. Additives are comparable to excipients for medicinal products and are often the same compounds [1]. The term “additives,” in contrast to “excipients” and “impurities,” does not appear in the German Medicines Act (AMG). Many excipients are characterized and specified in the European Pharmacopoeia (Pharm. Eur.; [2]). In contrast to the additives allowed in food, there is no closed list for excipients in medicinal products. New excipients must be characterized in detail by the drug manufacturers [3]. According to Pharm. Eur. 10.7, an excipient (auxiliary substance) is any constituent of a medicinal product that is not an active substance; adjuvants, stabilizers, antimicrobial preservatives, diluents, and antioxidants are examples of excipients [2]. The European Medicines Agency (EMA) Guideline on excipients in the marketing authorization application dossier (EMEA/CHMP/QWP/396951/2006; [3]) states: “Excipients include, e.g., fillers, disintegrants, lubricants, coloring matters, antioxidants, preservatives, adjuvants, stabilizers, thickeners, emulsifiers, solubilizers, permeation enhancers, flavoring and aromatic substances etc., as well as the constituents of the outer covering of the medicinal products, e.g., gelatin capsules.” All excipients must be listed in the Summary of Product Characteristics (SmPC) and the package leaflet of medicinal products for human use [3–5]. This also applies to natural rubber latex in the container of a medicinal product [5]. Residues that are of special relevance (e.g., ovalbumin in egg-derived vaccines) should be specified [6].

The availability, ingredients, and manufacturing processes of licensed vaccines, which have been summarized in various reviews [7, 8], vary in different countries and may also change over time. The contents of this review refer to the vaccines currently on the German market (Table 1), the content of the respective SmPCs, and the public part of the Medicinal Products and Application Database (*Arzneimittel- und Antrags-Datenbank* [AmAnDa]; https://portal.dimdi.de/amguifree/termsofuse.xhtml). The data were compiled and prepared with the utmost care and to the best of our knowledge; however, the authors do not assume any liability for the timeliness, correctness, completeness, or accuracy of the data. A list of vaccines approved in Germany and their respective ingredients can be found on the homepage of the Paul-Ehrlich-Institut and the current SmPCs linked there (https://www.pei.de/EN/medicinal-products/vaccines-human/vaccines-human-node.html; last accessed 4/3/2022).

**Table 1:** Potentially allergenic excipients, residues, and contaminants in vaccines* (as of 8 April 2022)

**Excipients**	**Contained in vaccines against**	**Products**
*Gelatin*	Influenza (nasal); Pandemic influenza (H5N1) (nasal) Typhoid (oral)	Fluenz Tetra 2021/2022 (AstraZeneca AB)^m^; Pandemic influenza vaccine H5N1 AstraZeneca^n^; Typhoral L (Emergent Netherlands B.V.)^ü^
*Hydrolyzed gelatin*	Shingles (Herpes zoster); Measles–mumps–rubella; Measles–mumps–rubella–varicella; Varicella	Zostavax (MSD VACCINS; Lyon; France)^i^; M‑M-RVAXPRO (MSD VACCINS)^p,s,x^; ProQuad (MSD VACCINS)^p,s,x,ö^; Varivax (Eurim-Pharm Arzneimittel GmbH)^ö^
*Polygelin*	Rabies	Rabipur (kohlpharma GmbH)^ä^
*Dextran*	Rotavirus	Rotarix (GlaxoSmithKline Biologicals S.A.)^y^
*Aluminum complexes (e.g., aluminum hydroxide or phosphate)*	Diphtheria–tetanus; Diphtheria–tetanus–pertussis; Diphtheria–tetanus–pertussis-poliomyelitis; Diphtheria–tetanus–pertussis–poliomyelitis–*Haemophilus influenzae* type B; Diphtheria—tetanus–pertussis–hepatitis B (recombinant)-poliomyelitis-*Haemophilus influenzae* type B; Tick-borne encephalitis (TBE); Human papillomavirus (HPV); Hepatitis A; Hepatitis A–hepatitis B (recombinant); Hepatitis A–typhus; Hepatitis B (recombinant), Japanese encephalitis; Group B meningococci (recombinant); Meningococcal serogroup C; Anthrax; Pneumococci	Td-IMMUN (AJ Vaccines A/S)^d,z^; Td-Impfstoff Merieux (Sanofi Pasteur Europe)^d,z^; Td-pur (AstroPharma GmbH)^d,z^; Boostrix (EurimPharm Arzneimittel GmbH)^d,t,z^; COVAXiS (Sanofi Pasteur Europe)^d,t,z^; Infanrix (GlaxoSmithKline GmbH & Co. KG)^d,t,z^; TdaP-IMMUN (AJ Vaccines A/S; Denmark)^d,t,z^; Boostrix Polio (EMRAMED Arzneimittel GmbH)^d,t,w,z^; Repevax (Sanofi Pasteur Europe)^d,t,w,z^; Revaxis (kohlpharma GmbH)^d,w,z^; Tetravac (EMRA-MED Arzneimittel GmbH)^d,t,w,z^; Infanrix-IPV + Hib (EMRA-MED Arzneimittel GmbH)^d,j,t,w,z^; Pentavac (EMRA-MED Arzneimittel GmbH)^d,j,t,w,z^; Hexacima (Sanofi Pasteur)^d,j,l,t,w,z^; Hexyon (Sanofi Pasteur)^d,j,l,t,w,z^; Infanrix hexa (GlaxoSmithKline Biologicals S.A.)^d,j,l,t,w,z^; Encepur Erwachsene (EMRA-MED Arzneimittel GmbH)^f^; Encepur Kinder (Bavarian Nordic A/S)^f^; FSME-IMMUN 0.5 ml Erwachsene (Pfizer Pharma GmbH)^f^; FSME-IMMUN 0.25 ml Junior (Pfizer Pharma GmbH)^f^; Cervarix (GlaxoSmithKline Biologicals S.A.)^g^; Gardasil (MSD VACCINS; Lyon; France)^g^; Gardasil 9 (MSD VACCINS; Lyon; France)^g^; Avaxim (Sanofi Pasteur Europe)^k^; Avaxim Junior (Sanofi Pasteur; F‑69007 Lyon)^k^; Havrix 720 Kinder (GlaxoSmithKline GmbH & Co. KG)^k^; Havrix 1440 (GlaxoSmithKline GmbH & Co. KG)^k^; VAQTA (kohlpharma GmbH)^k^; VAQTA Kinder (MSD Sharp & Dohme GmbH)^k^; Ambirix (GlaxoSmithKline Biological S.A.; Belgium)^k,l^; Twinrix Erwachsene (GlaxoSmithKline Biological S.A.; Belgium)^k,l^; Twinrix Kinder (GlaxoSmithKline Biological S.A.; Belgium)^k,l^; ViATIM (Sanofi Pasteur Europe)^k,ü^; Engerix‑B Erwachsene (HAEMATO PHARM GmbH)^l^; Fendrix (GlaxoSmithKline Biologicals S.A.)^l^; HBVaxPro (MSD VACCINS; Lyon; France)^l^; Ixiaro (Valneva Austria GmbH)^o^; Bexsero (GSK Vaccines S.r.l)^q^; Menjugate 10 μg (Pharma Gerke GmbH)^q^; NeisVac‑C (AxiCorp Pharma GmbH)^q^; Trumenba (Pfizer Limited; UK)^q^; BioThrax (Emergent Sales and Marketing Germany GmbH)^r^; Prevenar 13 (Pfizer Limited; UK)^u^; Synflorix (GlaxoSmithKline Biologicals S.A.)^u^; Vaxneuvance (Merck Sharp & Dohme B.V.; NL)^u^
*Phenoxyethanol; synonym: 2‑Phenoxyethanol*	Diphtheria–tetanus; Diphtheria–tetanus–pertussis; Diphtheria–tetanus–poliomyelitis; Diphtheria–tetanus–pertussis–Poliomyelitis; Diphtheria–tetanus–pertussis–poliomyelitis–*Haemophilus influenzae* type B; Hepatitis A; Hepatitis A–typhoid; Pneumococci; Poliomyelitis	Td-Impfstoff Merieux (Sanofi Pasteur Europe)^d,z^; COVAXiS (Sanofi Pasteur Europe)^d,t,z^; Revaxis (kohlpharma GmbH)^d,w,z^; Repevax (Sanofi Pasteur Europe)^d,t,w,z^; Tetravac (EMRA-MED Arzneimittel GmbH)^d,t,w,z^; Pentavac (EMRA-MED Arzneimittel GmbH)^d,j,t,w,z^; Avaxim (Sanofi Pasteur Europe)^k^; Avaxim Junior (Sanofi Pasteur; F‑69007 Lyon)^k^; ViATIM (Sanofi Pasteur Europe)^k,ü^; Prevenar 13 (Pfizer Limited; UK)^u^; Synflorix (GlaxoSmithKline Biologicals S.A.)^u^; Imovax Polio (EURIM-PHARM Arzneimittel GmbH)^w^; IPV Merieux (EMRAmed Arzneimittel GmbH)^w^
*Thiomersal*	Pandemic influenza (H5N1)	Adjupanrix (GlaxoSmithKline Biologicals S.A)^n^
*Polysorbate 80*	COVID-19; Diphtheria–tetanus–pertussis; Diphtheria–tetanus–poliomyelitis; Diphtheria–tetanus–pertussis–Poliomyelitis; Diphtheria–tetanus–pertussis–poliomyelitis–*Haemophilus influenzae* type B; Ebola vaccine (recombinant); Human papillomavirus vaccine (HPV); Zoster (recombinant); Hepatitis A; Hepatitis B (recombinant); Influenza; Pandemic influenza (H5N1); Meningococcal group B vaccine (recombinant); Pneumococci; Poliomyelitis; Rotavirus	Jcovden (formerly: COVID-19 Vaccine Janssen; Janssen-Cilag International NV)^b^; Nuvaxovid (NVX-CoV2373) (Novavax CZ a.s.)^b^; Vaxzevria (COVID-19 Vaccine AstraZeneca) (AstraZeneca AB; Sweden)^b^; Infanrix (GlaxoSmithKline GmbH & Co. KG)^d,t,z^; Revaxis (kohlpharma GmbH)^d,w,z^; Repevax (Sanofi Pasteur Europe)^d,t,w,z^; Infanrix-IPV + Hib (EMRA-MED Arzneimittel GmbH)^d,j,t,w,z^; Zabdeno (Janssen-Cilag International NV; Belgium)^l^; Gardasil (MSD VACCINS; Lyon; France)^g^; Gardasil 9 (MSD VACCINS; Lyon; France)^g^; Shingrix (GlaxoSmithKline Biologicals S.A.; Belgium)^i^; Avaxim (Sanofi Pasteur Europe)^k^; Avaxim Junior (Sanofi Pasteur; F‑69007 Lyon)^k^; Heplisav B (Dynavax GmbH)^l^; Flucelvax Tetra 2021/2022 (Seqirus Netherlands B.V.)^m^; Influsplit Tetra 2021/2022 (GlaxoSmithKline GmbH & Co. KG; Munich)^m^; Influvac Tetra 2021/2022 (Mylan Healthcare GmbH)^m^; Xanaflu Tetra 2021/2022 (Mylan Healthcare GmbH)^m^; Adjupanrix (GlaxoSmithKline Biologicals S.A)^n^; Trumenba (Pfizer Limited; UK)^q^; Prevenar 13 (Pfizer Limited; UK)^u^; Imovax Polio (EURIM-PHARM Arzneimittel GmbH)^w^; RotaTeq (MSD VACCINS)^y^
*Polyethylene glycol (PEG)*	Typhoid (oral)	Typhoral L (Emergent Netherlands B.V.)^ü^
*PEG (bound to lipid nanoparticles)*	COVID-19 (mRNA)	Comirnaty 30-μg concentrate for dispersion for injection (BioNTech Manufacturing GmbH); (supplied in vial with violet cap)^b^; Comirnaty 30-μg dispersion for injection (BioNTech Manufacturing GmbH); (supplied in vial with gray cap)^b^; Comirnaty 10-μg concentrate for dispersion for injection (BioNTech Manufacturing GmbH); (supplied in vial with orange cap)^b^; Spikevax (COVID-19 Vaccine Moderna) (Moderna Biotech Spain; S.L.)^b^
*Trometamol (TRIS)*	Diphtheria–tetanus–pertussis–hepatitis B (recombinant)–poliomyelitis–*Haemophilus influenzae *type B; Diphtheria–tetanus–pertussis–hepatitis B (recombinant)–poliomyelitis–*Haemophilus influenzae* type B; COVID-19 vaccine (mRNA); Dengue fever; Ebola Zaire; Ebola (recombinant); Tick-borne encephalitis (TBE); Haemophilus type B conjugate vaccine; Meningococcal groups A-; C‑; W135 and Y- Modified vaccinia virus Ankara Rabies vaccine (viral vaccine; inactivated)	Pentavac (EMRA-MED Arzneimittel GmbH) ^d,j,t,w,z^; Hexacima (Sanofi Pasteur)^d,j,l,t,w,z^; Hexyon (Sanofi Pasteur Europe)^d,j,l,t,w,z^; Comirnaty 30-μg dispersion for injection (BioNTech Manufacturing GmbH); (gray cap)^b^; Comirnaty 10-μg concentrate for dispersion for injection (BioNTech Manufacturing GmbH); (orange cap)^b^; Spikevax (COVID-19 Vaccine Moderna) (Moderna Biotech Spain; S.L.)^b^; Dengvaxia (Sanofi Pasteur; F‑69007 Lyon)^c^; Ervebo (Merck Sharp & Dohme B.V.)^e^ Mvabea (Janssen-Cilag International NV; Belgium)^e^; Encepur Erwachsene (EMRA-MED Arzneimittel GmbH)^f^; Encepur Kinder (Bavarian Nordic A/S)^f^; Act-HIB (EURIM-PHARM Arzneimittel GmbH)^j^; Nimenrix (Pfizer Limited; U.K.)^q^; Imvanex (Bavarian Nordic A/S)^v^; Rabipur (kohlpharma GmbH)^ä^
**Residues from manufacturing process**	**Contained in vaccines against**	**Products**
*Chicken egg white; Ovalbumin*	Ebola (recombinant); Tick-borne encephalitis (TBE); Yellow fever; Influenza (nasal); Influenza; Pandemic influenza (H5N1) (nasal); Pandemic influenza (H5N1); Measles–mumps–rubella; Measles–mumps–rubella–varicella; Modified vaccinia virus Ankara; Rabies	Mvabea (Janssen-Cilag International NV)^e^; Encepur Erwachsene (EMRA-MED Arzneimittel GmbH)^f^; Encepur Kinder (Bavarian Nordic A/S)^f^; FSME-IMMUN 0.5 ml Erwachsene (Pfizer Pharma GmbH)^f^; FSME-IMMUN 0.25 ml Junior (Pfizer Pharma GmbH)^f^; Stamaril (EMRA-MED Arzneimittel GmbH)^h^; Fluenz Tetra 2021/2022 (AstraZeneca AB)^m^; Afluria Tetra 2021/2022 (Seqirus Netherlands B.V.)^m^ Efluelda 2021/2022 (Sanofi Pasteur)^m^; Fluad Tetra 2021/2022 (Seqirus Netherlands B.V.)^m^; Influsplit Tetra 2021/2022 (GlaxoSmithKline GmbH & Co. KG; Munich)^m^; Influvac Tetra 2021/2022 (Mylan Healthcare GmbH)^m^; Vaxigrip Tetra 2021/2022 (Sanofi Pasteur)^m^; Xanaflu Tetra 2021/2022 (Mylan Healthcare GmbH)^m^; Pandemic influenza vaccine H5N1 AstraZeneca (AstraZeneca AB)^n^; Adjupanrix (GlaxoSmithKline Biologicals S.A)^n^; Priorix (A.C.A. Müller ADAG Pharma AG)^p,s,x^; Priorix-Tetra (GlaxoSmithKline GmbH & Co. KG)^p,s,x,ö^; Imvanex (Bavarian Nordic A/S)^v^; Rabipur (kohlpharma GmbH)^ä^
*Yeast fungal proteins (produced in yeast cells)*	Diphtheria–tetanus–pertussis–hepatitis B (recombinant)–poliomyelitis–*Haemophilus influenzae *type B; Hepatitis A–hepatitis B (recombinant); Hepatitis B (recombinant); Human papillomavirus vaccine (HPV)	Hexacima (Sanofi Pasteur)^d,j,l,t,w,z^; Hexyon (Sanofi Pasteur)^d,j,l,t,w,z^; Infanrix hexa (GlaxoSmithKline Biologicals S.A.)^d,j,l,t,w,z^; Vaxelis (MCM Vaccine B.V.)^d,j,l,t,w,z^; Twinrix Erwachsene (GlaxoSmithKline Biological S.A.; Belgium)^k,l^; Twinrix Kinder (GlaxoSmithKline Biological S.A.; Belgium)^k,l^; Ambirix (GlaxoSmithKline Biological S.A.; Belgium)^k,l^; Engerix‑B Erwachsene (HAEMATO PHARM GmbH)^l^; Fendrix (GlaxoSmithKline Biological S.A.)^l^; HBVaxPro (MSD VACCINS; Lyon; France)^l^; Heplisav B (Dynavax GmbH)^l^; Gardasil (MSD VACCINS; Lyon; France)^g^; Gardasil 9 (MSD VACCINS; Lyon; France)^g^
*Cow’s milk proteins; Casein; Lactalbumin*	Cholera (oral); Typhoid fever; Typhoid (oral)	Vaxchora (Emergent Netherlands B.V.)^a^; Typhim Vi (Sanofi Pasteur Europe)^ü^; Typhoral L (Emergent Netherlands B.V.)^ü^
*Human serum albumin (HSA)*	Ebola Zaire; Tick-borne encephalitis (TBE); Measles–mumps–rubella; Rabies	Ervebo (Merck Sharp & Dohme B.V.)^e^ FSME-IMMUN 0.5 ml Erwachsene (Pfizer Pharma GmbH)^f^ FSME-IMMUN 0.25 ml Junior (Pfizer Pharma GmbH)^f^ M‑M-RVAXPRO (MSD VACCINS)^p,s,z^ ProQuad (MSD VACCINS)^p,s,x,ö^ Rabipur (kohlpharma GmbH)^ä^ Tollwutimpfstoff (HDC) inaktiviert (Sanofi Pasteur Europe)^ä^
*Bovine serum albumin (BSA)*	Diphtheria–tetanus–pertussis-poliomyelitis; Diphtheria–tetanus–pertussis–hepatitis B (recombinant)–poliomyelitis (inactivated)–*Haemophilus influenzae* type B; Japanese encephalitis	Repevax (Sanofi Pasteur Europe)^d,t,w,z^; Vaxelis (MCM Vaccine B.V.)^d,j,l,t,w,z^; Ixiaro (Valneva Austria GmbH)^o^
*Porcine serum albumin*	–	–
*Peptone*	–	–
*Amphotericin B*	Rabies	Rabipur (kohlpharma GmbH)^ä^
*Chlortetracycline*	Tick-borne encephalitis (TBE); Rabies	Encepur Erwachsene (EMRA-MED Arzneimittel GmbH)^f^ Encepur Kinder (Bavarian Nordic A/S)^f^ Rabipur (kohlpharma GmbH)^ä^
*Neomycin*	Diphtheria–tetanus–poliomyelitis; Diphtheria–tetanus–pertussis–poliomyelitis; Diphtheria–tetanus–pertussis–poliomyelitis–*Haemophilus influenzae *type B; Diphtheria–tetanus–pertussis–hepatitis B (recombinant)–poliomyelitis (inactivated)–*Haemophilus influenzae* type B; Tick-borne encephalitis (TBE); Hepatitis A; Hepatitis A–hepatitis B (recombinant); Hepatitis A–typhus; Influenza; Measles–mumps–rubella; Measles–mumps–rubella–varicella; Poliomyelitis; Rabies; Varicella; Zoster	Revaxis (kohlpharma GmbH)^d,w,z^; Boostrix Polio (EMRA-MED Arzneimittel GmbH)^d,t,w,z^; Tetravac (EMRA-MED Arzneimittel GmbH)^d,t,w,z^; Infanrix-IPV + Hib (EMRA-MED Arzneimittel GmbH)^d,j,t,w,z^; Pentavac (EMRA-MED Arzneimittel GmbH) ^d,j,t,w,z^; Hexacima (Sanofi Pasteur)^d,j,l,t,w,z^; Hexyon (Sanofi Pasteur)^d,j,l,t,w,z^; Infanrix hexa (GlaxoSmithKline Biologicals S.A.)^d,j,l,t,w,z^; Vaxelis (MCM Vaccine B.V.)^d,j,l,t,w,z^; Encepur Erwachsene (EMRA-MED Arzneimittel GmbH)^f^; Encepur Kinder (Bavarian Nordic A/S)^f^; FSME-IMMUN 0.5 ml Erwachsene (Pfizer Pharma GmbH)^f^; FSME-IMMUN 0.25 ml Junior (Pfizer Pharma GmbH)^f^; Havrix 720 Kinder (GlaxoSmithKline GmbH & Co. KG)^k^; Havrix 1440 (GlaxoSmithKline GmbH & Co. KG)^k^; VAQTA (kohlpharma GmbH)^k^; VAQTA Kinder (MSD Sharp & Dohme GmbH)^k^; Ambirix (GlaxoSmithKline Biological S.A.; Belgium)^k,l^; Twinrix Erwachsene (GlaxoSmithKline Biological S.A.; Belgium)^k,l^; Twinrix Kinder (GlaxoSmithKline Biological S.A.; Belgium)^k,l^; ViATIM (Sanofi Pasteur Europe)^k,ü^; Afluria Tetra 2021/2022 (Seqirus Netherlands B.V.)^m^; Fluad Tetra 2021/2022 (Seqirus Netherlands B.V.)^m^; Vaxigrip Tetra 2021/2022 (Sanofi Pasteur)^m^; M‑M-RVAXPRO (MSD VACCINS)^p,s,x^; Priorix (A.C.A. Müller ADAG Pharma AG)^p,s,x^; Priorix-Tetra (GlaxoSmithKline GmbH & Co. KG)^p,s,x,ö^; ProQuad (MSD VACCINS)^p,s,x,ö^; Imovax Polio (EURIM-PHARM Arzneimittel GmbH)^w^; IPV Merieux (EMRAmed Arzneimittel GmbH)^w^; Rabipur (kohlpharma GmbH)^ä^; Tollwutimpfstoff (HDC) inaktiviert (Sanofi Pasteur Europe)^ä^; Varilrix (Eurim-Pharm Arzneimittel GmbH)^ö^; Varivax (Eurim-Pharm Arzneimittel GmbH)^ö^; Zostavax (MSD VACCINS; Lyon; France)^i^
*Gentamycin*	Diphtheria–tetanus–pertussis–poliomyelitis; Ebola (recombinant); Tick-borne encephalitis (TBE); Influenza (nasal); Influenza; Pandemic influenza (H5N1) (nasal); Pandemic influenza (H5N1); Modified vaccinia virus Ankara	Repevax (Sanofi Pasteur Europe)^d,t,23,z^; Mvabea (Janssen-Cilag International NV; Belgium)^e^; Encepur Erwachsene (EMRAMED Arzneimittel GmbH)^f^; Encepur Kinder (Bavarian Nordic A/S)^f^; FSME-IMMUN 0.5 ml Erwachsene (Pfizer Pharma GmbH)^f^; FSME-IMMUN 0.25 ml Junior (Pfizer Pharma GmbH)^f^; Fluenz Tetra 2021/2022 (AstraZeneca AB)^m^; Influsplit Tetra 2021/2022 (GlaxoSmithKline GmbH & Co. KG; Munich)^m^; Influvac Tetra 2021/2022 (Mylan Healthcare GmbH)^m^; Xanaflu Tetra 2021/2022 (Mylan Healthcare GmbH)^m^; Pandemic influenza vaccine H5N1 AstraZeneca (AstraZeneca AB)^n^; Adjupanrix (GlaxoSmithKline Biologicals S.A)^n^; Imvanex (Bavarian Nordic A/S)^v^
*Kanamycin*	Influenza	Fluad Tetra 2021/2022 (Seqirus Netherlands B.V.)^m^
*Polymyxin*	Diphtheria–tetanus–poliomyelitis; Diphtheria–tetanus–pertussis–poliomyelitis; Diphtheria–tetanus–pertussis–poliomyelitis–*Haemophilus influenzae *type B; Diphtheria–tetanus–pertussis–hepatitis B (recombinant)–poliomyelitis–*Haemophilus influenzae *type B; Influenza; Poliomyelitis	Revaxis (kohlpharma GmbH)^d,w,z^; Boostrix Polio (EMRA-MED Arzneimittel GmbH)^d,t,w,z^; Repevax (Sanofi Pasteur Europe)^d,t,w,z^; Tetravac (EMRA-MED Arzneimittel GmbH)^d,t,w,z^; Infanrix-IPV + Hib (EMRA-MED Arzneimittel GmbH)^d,j,t,w,z^; Pentavac (EMRA-MED Arzneimittel GmbH)^d,j,t,w,z^; Hexacima (Sanofi Pasteur)^d,j,l,t,w,z^; Hexyon (Sanofi Pasteur)^d,j,l,t,w,z^; Infanrix hexa (GlaxoSmithKline Biologicals S.A.)^d,j,l,t,w,z^; Vaxelis (MCM Vaccine B.V.)^d,j,l,t,w,z^; Afluria Tetra 2021/2022 (Seqirus Netherlands B.V.)^m^; Imovax Polio (EURIM-PHARM Arzneimittel GmbH)^w^; IPV Merieux (EMRAmed Arzneimittel GmbH)^w^
*Streptomycin*	Diphtheria–tetanus–poliomyelitis; Diphtheria–tetanus–pertussis–poliomyelitis; Diphtheria–tetanus–pertussis–poliomyelitis–*Haemophilus influenzae* type B; Diphtheria–tetanus–pertussis–hepatitis B (recombinant)-poliomyelitis–*Haemophilus influenzae *type B; Influenza; Poliomyelitis	Revaxis (kohlpharma GmbH)^d,w,z^; Repevax (Sanofi Pasteur Europe)^d,t,w,z^; Tetravac (EMRA-MED Arzneimittel GmbH)^d,t,w,z^; Pentavac (EMRA-MED Arzneimittel GmbH)^d,j,t,w,z^; Hexacima (Sanofi Pasteur)^d,j,l,t,w,z^; Hexyon (Sanofi Pasteur)^d,j,l,t,w,z^; Vaxelis (MCM Vaccine B.V.)^d,j,l,t,w,z^; Imovax Polio (EURIM-PHARM Arzneimittel GmbH)^w^; IPV Merieux (EMRAmed Arzneimittel GmbH)^w^
*Formaldehyde*	Diphtheria–tetanus; Diphtheria–tetanus–pertussis; Diphtheria–tetanus–poliomyelitis; Diphtheria–tetanus–pertussis–poliomyelitis; Diphtheria–tetanus–pertussis–poliomyelitis–*Haemophilus influenzae *type B; Diphtheria–tetanus–pertussis–hepatitis B (recombinant)-poliomyelitis–*Haemophilus influenzae *type B; Tick-borne encephalitis (TBE); *Haemophilus influenzae* type B; Hepatitis A; Hepatitis B (recombinant); Influenza; Pandemic influenza (H5N1); Japanese encephalitis; Anthrax; Poliomyelitis; Typhoid	Td-Impfstoff Merieux (Sanofi Pasteur Europe)^d,z^; Td-pur (Astro-Pharma GmbH)^d,z^; COVAXiS (Sanofi Pasteur Europe)^d,t;z^; TdaP-IMMUN (AJ Vaccines A/S; Denmark)^d,t;z^; Revaxis (kohlpharma GmbH)^d,w,z^; Repevax (Sanofi Pasteur Europe)^d,t,w,z^; Tetravac (EMRA-MED Arzneimittel GmbH)^d,t,w,z^; Pentavac (EMRA-MED Arzneimittel GmbH)^d,j,t,w,z^; Hexacima (Sanofi Pasteur)^d,j,l,t,w,z^; Hexyon (Sanofi Pasteur)^d,j,l,t,w,z^; Infanrix hexa (GlaxoSmithKline Biologicals S.A.)^d,j,l,t,w,z^; Vaxelis (MCM Vaccine B.V.)^d,j,l,t,w,z^; Encepur Erwachsene (EMRA-MED Arzneimittel GmbH)^f^; Encepur Kinder (Bavarian Nordic A/S)^f^; FSME-IMMUN 0.5 ml Erwachsene (Pfizer Pharma GmbH)^f^; FSME-IMMUN 0.25 ml Junior (Pfizer Pharma GmbH) ^f^; Act-HIB (EURIM-PHARM Arzneimittel GmbH)^j^; Avaxim (Sanofi Pasteur Europe)^k^; Avaxim Junior (Sanofi Pasteur; F‑69007 Lyon)^k^; VAQTA (kohlpharma GmbH)^k^; VAQTA Kinder (MSD Sharp & Dohme GmbH)^k^; HBVaxPro (MSD VACCINS; Lyon; France)^l^; Efluelda 2021/2022 (Sanofi Pasteur)^m^; Fluad Tetra 2021/2022 (Seqirus Netherlands B.V.)^m^; Influsplit Tetra 2021/2022 (GlaxoSmithKline GmbH & Co. KG; Munich)^m^; Influvac Tetra 2021/2022 (Mylan Healthcare GmbH)^m^; Vaxigrip Tetra 2021/2022 (Sanofi Pasteur)^m^; Xanaflu Tetra 2021/2022 (Mylan Healthcare GmbH)^m^; Adjupanrix (GlaxoSmithKline Biologicals S.A)^n^; Ixiaro (Valneva Austria GmbH)^o^; BioThrax (Emergent Sales and Marketing Germany GmbH)^r^; Imovax Polio (EURIM-PHARM Arzneimittel GmbH)^w^; IPV Merieux (EMRAmed Arzneimittel GmbH)^w^; Typhim Vi (Sanofi Pasteur Europe)^ü^
*Glutaraldehyde*	Diphtheria–tetanus–pertussis; Diphtheria–tetanus–pertussis–poliomyelitis; Diphtheria–tetanus–pertussis–poliomyelitis–*Haemophilus influenzae *type B; Diphtheria–tetanus–pertussis–hepatitis B (recombinant)–poliomyelitis–*Haemophilus influenzae* type B	COVAXiS (Sanofi Pasteur Europe)^d,t;z^; Repevax (Sanofi Pasteur Europe)^d,t,w,z^; Tetravac (EMRA-MED Arzneimittel GmbH)^d,t,w,z^; Pentavac (EMRA-MED Arzneimittel GmbH) ^d,j,t,w,z^; Hexacima (Sanofi Pasteur)^d,j,l,t,w,z^; Hexyon (Sanofi Pasteur)^d,j,l,t,w,z^; Vaxelis (MCM Vaccine B.V.)^d,j,l,t,w,z^
**Contamination**	**Potentially contained in vaccines against**	**Product**
*Natural rubber latex*	Hepatitis A	VAQTA (kohlpharma GmbH)^k^

*For manufacturer’s detailed information (company, city, country) please visit: https://www.pei.de/EN/medicinal-products/vaccines-human/vaccines-human-node.html.

Specific manufacturing methods (recombinant; mRNA) and routes of administration (oral; nasal) in parentheses. Vaccine against:

^a^Cholera

^b^COVID-19

^c^Dengue fever

^d^Diphtheria

^e^Ebola

^f^Tick-borne encephalitis (TBE)

^g^Human papillomavirus (HPV)

^h^Yellow fever

^i^Shingles (zoster)

^j^*Haemophilus influenzae* type B

^k^Hepatitis A

^l^Hepatitis B

^m^Influenza

^n^Pandemic influenza (H5N1)

^o^Japanese encephalitis

^p^Measles

^q^Meningococcus

^r^Anthrax

^s^Mumps

^t^Pertussis

^u^Pneumococci

^v^Smallpox

^w^Poliomyelitis

^x^Rubella

^y^Rotavirus

^z^Tetanus

^ä^Rabies

^ü^Typhoid

^ö^Chickenpox (varicella)

## Anaphylaxis to vaccines

The occurrence of an anaphylactic reaction cannot be excluded in principle after vaccination, but it represents a very rare event (one to ten cases per 1 million vaccinations); this applies equally to the vaccines in pre-pandemic use [8–11] and to the COVID-19 vaccines [12, 13]. The still widely cited 11.1 cases of anaphylaxis per 1 million first doses of Comirnaty administered, observed at the start of the vaccination campaign in the United States [14], had already declined to 4.7 cases per 1 million doses of vaccine administered by the second week of the vaccination campaign [12]. Over- and under-reporting must be considered in spontaneous reporting registries. The frequency of confirmed anaphylaxis cases (Brighton Collaboration [BC] level 1–3 [15]) reported in Germany in connection with the administration of COVID-19 vaccines in the spontaneous reporting register in relation to the number of vaccine doses administered between 27 December 2020 and 31 December 2021 [13] was for Comirnaty (BioNTech/Pfizer) approximately 2.0 per 1 million doses, for Spikevax (MODERNA) approximately 0.8 per 1 million doses, for Vaxzevria (AstraZeneca) approximately 3.5 per 1 million doses, and for Jcovden (formerly: COVID-19 vaccine Janssen) approximately 1.1 per 1 million doses. However, no direct comparison is possible due to confounders in spontaneous reporting registers.

In addition to immunoglobulin (Ig)E-mediated anaphylaxis, non-IgE-mediated anaphylaxis (formerly referred to as “anaphylactoid reactions”) may occur. These may be due to direct release of certain messenger substances without the presence of IgE antibodies [9]. Accidental injection into a blood vessel (intravascular administration) may also be considered as a cause. Anaphylactoid reactions after vaccination are very rare [9]. In contrast to IgE-mediated anaphylactic reactions, they can also occur during a first vaccination (without previous contact with the allergen [9]).

Both anaphylactic and anaphylactoid reactions must be distinguished from functional circulatory disturbances in response to the injection (so-called vasovagal reactions). It can be assumed that the frequency of such reactions corresponds to that of collapse states after other injections [9].

## Triggers of anaphylaxis to vaccines (before COVID-19)

Potential triggers of IgE-mediated reactions in different vaccines include:

- The vaccine antigens themselves (e.g., pertussis toxin, tetanus toxin)
- Excipients (e.g., gelatin derivatives, dextran)
- Residues (traces from the manufacturing process, e.g., chicken egg protein, antibiotics, formaldehyde, yeast proteins, cow milk proteins, serum proteins)
- Contamination due to vial stopper of multi-dose containers (e.g., natural rubber latex stopper)

### Excipients with a known allergenic risk potential

#### Gelatin and polygeline

Gelatin is a type I allergen found in many foods. Gelatin is/was added to both live and inactivated vaccines as a stabilizing agent [8, 9]. It has previously caused systemic allergic reactions as a component of some acellular pertussis vaccines, with one third of patients exhibiting IgE antibodies to gelatin [9]. Gelatin is currently contained only in a few vaccines (see Table 1).

The gelatin derivative polygeline (a polymer of degraded gelatin with urea bridges) has been used as a stabilizer in various vaccines (e.g., rabies, MMR, and varicella vaccines; [9]) and is currently only present in the rabies vaccine Rabipur®. Polygeline can also cause type I allergies [9]. It is known from infusion solutions for use in blood loss (which contain polygeline in large quantities, such as Haemaccel®) that polygeline can also cause direct histamine release; in a few patients (about 1%), this results in pseudoallergic reactions, mainly manifesting as skin reactions [9].

A polygeline-containing TBE vaccine for children was withdrawn from the market in 1998 due to anaphylactic reactions following its administration. Subsequently, the vaccine was developed polygeline-free and reintroduced to the market in this form in 2001. A comparative review of adverse reaction for this vaccine before and after the stabilizer polygeline was removed clearly showed that the reporting rate of anaphylactic reactions per vaccination thereafter returned to normal background levels for vaccinations [9]. In addition, IgE antibodies to gelatin were found in 12 of 14 children with allergic reactions to the polygeline-containing vaccine [9].

#### Dextran

Dextran is occasionally used in vaccines as a nutrient medium or stabilizer. During the national MMR vaccination campaign in Brazil in 2004, the rate of hypersensitivity reactions after MMR vaccination was unexpectedly high [16]. Dextran was identified as a likely cause of these hypersensitivity reactions [16]. The only vaccine in Germany currently containing dextran is the rotavirus vaccine (live, attenuated) Rotarix®.

#### Other allergenic excipients that may be present in vaccines

- Aluminum complexes, e.g., aluminum hydroxide or phosphate; currently included as adjuvants in numerous vaccines (see Table 1)
- Phenoxyethanol; contained as a preservative in various vaccines (see Table 1)
- Thiomersal; preservative; currently used in Germany exclusively as an ingredient in the multi-dose container of Adjupanrix®, a pandemic influenza vaccine (H5N1; split virion, inactivated, adjuvanted) and in immunostimulants against bacterial urinary tract infections (Strovac®, Booster-Strovac® and Perison®)

These three excipients mentioned are known contact allergens that can very rarely cause (also generalized) contact dermatitis in connection with vaccinations. They do *not have any* anaphylactogenic potential.

A specific investigation of patients with known contact allergy to thiomersal showed that intramuscular vaccination with a thiomersal-containing vaccine did not cause contact allergic reactions in the majority of them [9].

### Residues in drugs with an allergenic risk potential

#### Hen’s egg and chicken protein

In the pre-COVID-19 era, a known hen’s egg allergy represented a regularly occurring reason for seeking allergology consultation prior to vaccinations. Most vaccines, for example, single vaccines against hepatitis A and B and combination vaccines for basic immunization of infants and young children (against diphtheria, pertussis, tetanus, hepatitis B, polio, *Haemophilus influenzae* type B), do not contain chicken protein and are therefore not problematic for individuals with hen’s egg allergy. Vaccines against MMR (measles, mumps, rubella), MMRV (measles, mumps, rubella, varicella [chickenpox]), yellow fever, tick-born encephalitis (TBE), and influenza (flu) may contain traces of chicken protein. According to the European Pharmacopoeia [2], an upper limit of 1 µg ovalbumin per vaccine dose must not be exceeded, with the exception of yellow fever vaccines, where an upper limit of 5 µg ovalbumin per vaccine dose must not be exceeded [2].

A distinction must be made between vaccines in which the virus is grown on chicken embryos (influenza, yellow fever) and vaccines in which the virus is grown on chicken fibroblasts (MMR, rabies, TBE). The latter contain at most very small, barely detectable traces of egg protein with no allergenic potency [17]. International studies show that even children with a known history of hen’s egg allergy can be vaccinated against measles, mumps, and rubella without any problems or risk [17]. Hen’s egg allergy is no longer mentioned as a contraindication in international and national guidelines [17]. Exclusively children with clinically very severe egg protein allergy (e.g., anaphylactic shock after consumption of minute amounts of hen’s egg protein) should be vaccinated under special protective measures and subsequent observation (in hospital if necessary [17]). For MMR vaccines and rabies vaccine Rabipur®, hen’s egg protein allergy is mentioned under special warnings and precautions for use. In the SmPCs for TBE vaccines, the information on egg protein allergy varies product-specifically between warning and contraindication. This is due to the fact that pharmaceutical companies have the discretion to provide more extensive contraindications or warnings in the SmPC in order to ensure that their products are used safely than would be necessary according to medical guidelines. Under the terms of medical law, the content of the product-specific SmPC applies in each case.

For influenza and yellow fever vaccines, hen’s egg and chicken protein allergy is listed as a contraindication in the product information. However, a large number of clinical studies indicate that serious allergic reactions to influenza vaccination are rare even in persons with hen’s egg allergy, or do not occur more frequently than in individuals without hen’s egg allergy [18, 19]. Even in influenza vaccines prepared using chicken eggs, the egg protein content is usually below the dose that usually causes reactions [20]. Individuals who react with mild symptoms upon the consumption of hen’s egg protein can be vaccinated with all licensed influenza vaccines [21]. According to the recommendation of the Center of Disease Control (CDC) in the United States, no special surveillance measures are required [22]. Clinically severe allergies (e.g., anaphylaxis) to hen’s egg white are rare [21]. In individuals with a physician-diagnosed severe allergy to egg protein, the indication for vaccination with chicken egg-based influenza vaccines should be restrictive [20]. These individuals should be vaccinated in a medical setting where clinical monitoring after vaccination and treatment of any anaphylactic reaction that may occur are possible [21, 22]. In Germany, a chicken egg protein-free influenza vaccine from cell cultures (Flucelvax Tetra®) is available as an alternative.

The situation is different for yellow fever vaccination, which has egg protein concentrations that can lead to symptoms in approximately 5% of egg allergic patients [20]. In cases of hen’s egg allergy, the indication should be reserved and only given in cases of extreme necessity [20]. Yellow fever vaccine is administered only in designated vaccination centers by vaccination specialists. If an indication exists despite chicken egg allergy, vaccination should be given under special supervision in day-care, clinical, or outpatient facilities with the possibility of emergency intervention [20].

A comprehensive written explanation regarding risks and side effects and the contraindication outlined in the SmPC and written patient´s consent is required in the case of chicken egg protein allergy (“off-label use”).

#### Antibiotics

The antibiotics used today in vaccine production (neomycin, polymyxin B, kanamycin, gentamicin, chlortetracycline, framycetin, streptomycin residues from stock cultures) are those that are not preferentially used clinically in Germany [9]. As a component in vaccines (see Table 1), they therefore represent a rather low allergenic risk, although type I allergies have been described against many of these substances, so that caution is certainly advisable if such sensitization is actually present [9]. Known allergies in the past medical history to antibiotics contained in vaccines are listed in the majority of the SmPCs of vaccines under section 4.3 (Contraindications), for a few vaccines under section 4.4 (Special warnings and precautions for use), which must be taken into account in the informed patient consent. Where deemed appropriate, switching to a vaccine without this ingredient or vaccination under special clinical monitoring conditions is feasible [9]. Penicillin and streptomycin are not used at any stage of manufacture and are not added to the final product; however, stock cultures prepared with media containing penicillin or streptomycin may be used in justified and approved cases for manufacture according to the European Pharmacopoeia (Monograph 0153—“Vaccines for human use” [2]). Vaccines on the market in Germany do not contain penicillin and cephalosporin antibiotics. Persons with corresponding type I allergies to beta-lactam antibiotics can therefore be vaccinated without concern [9].

Type IV allergy to neomycin or structurally similar aminoglycoside antibiotics (framycetin, kanamycin, streptomycin) is not considered a contraindication to vaccination with a neomycin-containing vaccine [9].

#### Formaldehyde and glutaraldehyde

Formaldehyde and, less frequently, glutaraldehyde (see Table 1) are used in the production of some viral and bacterial vaccines to inactivate the source materials (bacterial toxins, viruses; [9]). The European Pharmacopoeia (Monograph 0153—“Vaccines for human use”) limits the residual content of formaldehyde in vaccines to 0.2 g/l, which corresponds to a concentration of 0.02% [2].

Individuals with specific IgE antibodies to formaldehyde (or to adducts of formaldehyde and endogenous proteins) have been described in the literature [9]. Clinical reactions, such as hives and even anaphylactic reactions, have been described in connection with dental products or the use of disinfectant solutions [9]. However, most reactions tend to be anaphylactoid (non-IgE-mediated, pseudoallergic) in nature, and formaldehyde is not considered a clinically significant type I sensitizer overall. An IgE-mediated anaphylactic reaction to vaccination in the presence of proven type I allergy to formaldehyde has not been reported [8, 9].

Formaldehyde is of far greater importance as a contact allergen, but the prevalence of formaldehyde contact allergy has declined remarkably since the 1980s due to the substantial decrease in industrial applications, surface disinfection, and cosmetics [9]. Although formaldehyde is present as a residual only in small quantities in vaccine preparations, the occurrence of generalized formaldehyde-specific contact dermatitis has been reported casuistically after administration of a formaldehyde-containing influenza vaccine [23]. Glutaraldehyde is also known as a contact allergen (e.g., when used as a surface disinfectant), but a manifestation of contact allergy due to glutaraldehyde residues in vaccines has not been published to date, as far as the authors are aware.

#### Human albumin

Cases of a generalized allergic reaction or even shock symptoms have not yet been reported in connection with human serum albumin (HSA) in vaccines (see Table 1). HSA-containing Ebola vaccine Ervebo® is currently not marketed in Germany.

Administration of larger amounts of human albumin in the form of infusions can lead to type III reactions according to Coombs and Gell classification (reactions due to immune complexes of antigen and antibodies), which, however, are rather unlikely to occur with the small amounts of albumin in vaccines [9].

#### Bovine serum albumin

Bovine serum albumin (BSA) may be present in trace amounts as a residual component from manufacturing in the vaccines Repevax®, Vaxelis®, and Ixiaro® available in Germany (see Table 1). IgE-mediated reactions against BSA have been associated with a case series of immediate-type reactions against various vaccines containing bovine/porcine adjuvants in Sri Lanka [24]. Anaphylaxis with detection of BSA-specific IgE antibodies has been reported casuistically in association with the use of BSA-containing cell culture media in artificial insemination [25, 26], as well as tumor vaccination with human peptide-pulsed dendritic cells [27].

#### Cow’s milk proteins (casein or alpha-lactalbumin)

Milk proteins can be used as growth media in diphtheria–tetanus–pertussis (DTaP and Tdap) vaccines [8, 28]. Bovine casein has been detected in nanogram amounts in these vaccines [28]. A case series of eight children with severe cow’s milk allergy who reacted with anaphylaxis to the booster dose of DTaP or Tdap vaccine has been reported, and casein contained in the vaccines has been discussed as a potential trigger [28, 29]. The methods used in this report have been controversial [28, 29]. According to an EAACI position paper, vaccination with DTaP and Tdap vaccines does not contribute to the pathogenesis of allergic diseases, and atopy is not a contraindication for the use of these vaccines [30].

Casein is mentioned as an ingredient in only a few of the vaccines marketed in Germany (see Table 1): Hydrolyzed casein is listed as an excipient in the SmPC of cholera vaccine Vaxchora® (effervescent powder and powder for the preparation of an oral suspension) and represents a contraindication in the case of allergy. The typhoid vaccines Typhim Vi® and Typhoral L® may contain traces of casein from the manufacturing process; an existing casein hypersensitivity is listed as a warning in the SmPC.

#### Yeast proteins (*Saccharomyces cerevisiae*)

Many vaccines contain antigens generated in cell lines [8]. Hepatitis B vaccines (including combination vaccines containing the hepatitis B surface antigen [HbsAg]) and some vaccines (Gardasil/Silgard, Gardasil 9) against human papillomavirus (HPV) contain recombinant antigens expressed in Baker’s yeast (*Saccharomyces cerevisiae*). Purification removes most of the cellular material, but it is not possible to remove all traces [8]. Between 1990 and 2004, only 15 reports of probable or possible anaphylaxis following vaccination of individuals with a history of yeast allergy were identified [8]. Eleven of these cases occurred after administration of a hepatitis B vaccine containing trace amounts of yeast proteins. Because these individuals were not tested for yeast allergy, it cannot be confirmed that a type I allergy to yeast proteins caused these adverse reactions, but the data suggest that the recombinant hepatitis B vaccine carries a minimal risk of allergic reactions in yeast-sensitive individuals [8].

According to a pharmacovigilance report from the United States, 107 adverse events had been reported in the Vaccine Adverse Event Reporting System (VAERS) there in individuals with a history of yeast allergy; of these, probable or possible anaphylaxis occurred in 11 recipients of hepatitis B vaccine [30]. By contrast, another study found no anaphylaxis events in a large cohort of women who had a positive skin test for yeast extract after HPV vaccination [31]. In another study investigating cases of anaphylxis following the quadrivalent human papillomavirus vaccination in Australia, all tested individuals had negative reactions to skin-prick testing for baker’s yeast, and to skin-prick testing and intradermal testing for Gardasil, Cervarix and polysorbate 80 [32].

In the case of the aforementioned vaccines, yeast cells are mentioned in the technical information as the expression systems of the active substances; residues of yeast proteins are not listed.

## Anaphylaxis to COVID-19 vaccines

mRNA-COVID-19 vaccines are based on a new manufacturing technique that has not yet been used in other vaccines. They contain novel, partially PEGylated lipid nanoparticles in which polyethylene glycol is present in covalent bound form. COVID-19 vector vaccines are also a relatively new manufacturing technique that has only been used in a few vaccines before (e.g., against Ebola and dengue fever). Table 2 provides an overview of the ingredients of the five COVID-19 vaccines currently licensed in Germany. Different vaccine components are potential triggers of anaphylaxis, whereby there may be different (IgE-mediated or non-IgE-mediated) underlying pathophysiological mechanisms.

**Table 2:** Ingredients of COVID-19 vaccines (as of 8 April 2022)

Trade name, *Company*, Vaccine type	Active ingredient	Excipients
**Comirnaty 30** **microgram/dose concentrate for dispersion for injection (12 years and older)—purple cap,** *BioNTech-Pfizer,* **mRNA **(embedded in lipid nanoparticles)	**BNT162b2** **=Tozinameran**	**((4-hydroxybutyl)azanediyl)bis(hexane‑6,1‑diyl)bis(2-hexyl decanoate) (ALC-0315)**
		**2-[(polyethylene glycol)-2000]-N,N-ditetradecylacetamide (ALC-0159)**
		**1,2-Distearoyl-sn-glycero-3-phosphocholine (DSPC)**
		**Cholesterol**
		Potassium chloride
		Potassium dihydrogen phosphate
		Sodium chloride
		Disodium phosphate dihydrate Sucrose
		Water for injections
		Sodium hydroxide (for pH adjustment)
		Hydrochloric acid (for pH adjustment)
**Comirnaty 30** **μg/dose dispersion for injection (12 years and older) “ready-to-use”-formulation—gray cap** *BioNTech-Pfizer,* **mRNA **(embedded in lipid nanoparticles)	**BNT162b2** **=Tozinameran**	**((4-Hydroxybutyl)azanediyl)bis(hexane‑6,1‑diyl)bis(2-hexyl decanoate) (ALC-0315)**
		**2-[(polyethylene glycol)-2000]-N,N-ditetradecylacetamide (ALC-0159)**
		**1,2-Distearoyl-sn-glycero-3-phosphocholine (DSPC)**
		**Cholesterol**
		**Trometamol**
		**Trometamol hydrochloride**
		Sucrose
		Water for injections
**Comirnaty 10** **μg/dose concentrate for dispersion for injection (children 5–11 years)—orange cap,** *BioNTech-Pfizer,* **mRNA **(embedded in lipid nanoparticles)	**BNT162b2** **=Tozinameran**	**((4-Hydroxybutyl)azanediyl)bis(hexane‑6,1‑diyl)bis(2-hexyl decanoate) (ALC-0315)**
		**2-[(polyethylene glycol)-2000]-N,N-ditetradecylacetamide (ALC-0159)**
		**1,2-Distearoyl-sn-glycero-3-phosphocholine (DSPC)**
		**Cholesterol**
		**Trometamol**
		**Trometamol hydrochloride**
		Sucrose
		Water for injections
**Spikevax (formerly: COVID-19 Vaccine Moderna) dispersion for injection,** *Moderna,* **mRNA **(embedded in SM-102 lipid nanoparticles)	**mRNA-1273**	**Lipid SM-102**^**d**^
		**Cholesterol**
		**1,2-Distearoyl-sn-glycero-3-phosphocholine (DSPC)**
		**1,2-Dimyristoyl-rac-glycero-3-methoxypolyethylene glycol-2000 (PEG2000 DMG)**
		**Trometamol**
		**Trometamol hydrochloride**
		Acetic acid
		Sodium acetate trihydrate
		Sucrose
		Water for injections
**Vaxzevria (formerly: COVID-19 Vaccine AstraZeneca) suspension for injection,** *AstraZeneca,* **Vector vaccine: **Chimpanzee Adenovirus encoding the SARS-CoV‑2 Spike glycoprotein (ChAdOx1-S)^a^	**AZD1222**	L‑Histidine
		L‑histidine hydrochloride monohydrate
		Magnesium chloride hexahydrate
		**Polysorbate-80**
		Ethanol
		Sucrose
		Sodium chloride
		Disodium edetate dihydrate
		Water for injections
**Jcovden (formerly: COVID-19 Vaccine Janssen) suspension for injection,** *Janssen-Cilag,* **Vector vaccine**: adenovirus type 26 encoding the SARS-CoV‑2 spike glycoprotein^b^ (Ad26.COV2-S)	**Ad26.COV2.S**	2‑hydroxypropyl-β-cyclodextrin (HBCD)
		Citric acid monohydrate
		Ethanol
		Hydrochloric acid
		**Polysorbate-80**
		Sodium chloride
		Sodium hydroxide
		Trisodium citrate dihydrate
		Water for injections
**Nuvaxovid dispersion for injection,** *Novavax,* **protein-based vaccine, virus-like particles (spike protein)** One dose (0.5 ml) contains 5 μg of the SARS-CoV‑2 spike protein^c^ and is adjuvanted with Matrix‑M. Adjuvant Matrix‑M contains per 0.5 ml dose: Fraction‑A (42.5 μg) *and Fraction‑C (7.5* *μg) of Quillaja saponaria* Molina extract	**NVX-CoV2373**	Disodium hydrogen phosphate 7 H2O
		Sodium dihydrogen phosphate 1 H2O
		Sodium chloride
		**Polysorbate 80**
		Sodium hydroxide (to adjust the pH value)
		Hydrochloric acid (to adjust the pH value)
		Water for injections
		*Adjuvant (Matrix-M):*
		**Cholesterol**
		**Phosphatidylcholine **(including all-rac-α-tocopherol)
		Potassium dihydrogen phosphate
		Potassium chloride
		Disodium hydrogen phosphate 2 H2O
		Sodium chloride
		Water for injections

Ingredients potentially involved in inducing anaphylaxis are in bold

^a^Produced in genetically modified human embryonic kidney (HEK) 293 cells and by recombinant DNA technology

^b^Produced in the PER.C6 TetR cell line (genetically modified human primary embryonic retinoblasts) and by recombinant DNA technology

^c^Produced by recombinant DNA technology using a baculovirus expression system in an insect cell line derived from Sf9 cells of the *Spodoptera frugiperda *species

^d^Synonym: heptadecan-9-yl-8-{(2-hydroxyethyl)[6-oxo-6-(undecyloxy)hexyl]amino}octanoate (IUPAC)

The following ingredients may constitute potential triggers of anaphylaxis:

- **Polyethylene glycol (PEG in PEGylated lipid nanoparticles)**
  - PEG-specific IgE has been described in the context of other drugs [33–37];
  - Anaphylactic reactions to PEG in drugs are known [38, 39].
- **Phospholipid (DSPC)**
  - Specific IgE and positive prick tests for phospholipids have been described [40].
- **Trometamol **(Tris): isolated cases of IgE-reactions have been published [41].
- **Particles: “CARPA” (complement-activation related pseudo allergy). **Pseudoallergic hypersensitivity reactions after intravenous administration of colloidal drugs have been reported in connection with various drugs (contrast media, liposomal drugs, nanoparticles; [42–44]).
- **Individual lipids (cholesterol, phospholipid [DSPC]):**
  - Non-PEGylated lipids can also trigger non-IgE-mediated mast cell degranulation [45].
- **Polysorbate 80 (Tween)**: isolated cases of IgE- and non-IgE-mediated reactions have been described [37, 46].

Which mechanisms play a role in the individual case may vary. While IgE directed against PEG has been plausibly described in individual cases [47], this does not seem to be the etiopathogenetically relevant mechanism in the majority of cases. Arguing against an IgE-mediated mechanism is the fact that the majority of anaphylactic reactions occurred at the first administration [13], the majority of cases with previous anaphylaxis to an mRNA-COVID-19 vaccine tolerated re-administration of an mRNA-COVID-19 vaccine under surveillance [48], and in a well-studied case series of 22 individuals with allergic reactions to an mRNA-COVID-19 vaccine, no PEG-specific or polysorbate 80-specific IgE was detectable, but PEG-specific IgG was detectable, suggesting a non-IgE-mediated mechanism such as CARPA [49].

## Known allergies to excipients and COVID-19 vaccination

On the first day of the vaccination campaign conducted in the United Kingdom with the mRNA-based vaccine Comirnaty from BioNTech/Pfizer, which was already being used there with a time-limited emergency use approval before the EMA rolling review was completed, the first two cases of a severe allergic reaction were reported. Such reactions were not known from the pivotal studies involving more than 40,000 participants. Based on the individual history of these two cases, the UK Medicines and Healthcare Products Regulatory Agency (MHRA) issued as guidance on 9 December 2020, “Any person with a history of anaphylaxis to a vaccine, medicine or food should not receive the Pfizer/BioNTech vaccine.” This opinion was withdrawn without restriction by the agency as early as 30 December 2020, after review of additional data [50].

Hypersensitivity to the active ingredient or any of the other ingredients of COVID-19 vaccine listed in Section 6.1 (“List of excipients”) of the respective SmPC (Table 2) is a contraindication to administration.

By contrast, other known allergies do not represent a contraindication, including allergies to drugs, antibiotics, and chicken protein [51]. The approach to pre-existing allergies and COVID-19 vaccination has been summarized in a consensus flow chart [51], which is also published on the homepages of PEI, DGAKI, and AEDA.

Despite the very rare incidence of anaphylactic reactions following COVID-19 vaccination, consistent 15-min monitoring of all vaccinees—with and without a history of allergy—is required.

The occurrence of delayed local reactions, including delayed skin reactions, has been reported in some cases after vaccination with both Spikevax and Comirnaty, and occasionally after vaccination with Vaxzevria, the AstraZeneca COVID-19 vaccine. In the United States and Canada, this reaction is referred to as “COVID arm.” The exact mechanism is not known, but the time interval from vaccination and the course suggests a delayed cutaneous hypersensitivity reaction (type IV) associated with the endogenous immune response [52]. It subsides spontaneously after a few days. The reaction does not constitute a reason to suspend or delay the second vaccination dose in affected individuals [52].

## Notes

The authors state that the contents and positions expressed in this review article reflect the personal expert opinion of the authors and should not be interpreted or quoted as if they had been commissioned by or reflected the position of the competent national higher federal authority, the European Medicines Agency, or any of their committees or working groups.

## Abbreviations

AmAnDa: Medicinal Products and Application Database
BC: Brighton Collaboration
BSA: Bovine serum albumin
CARPA: Complement-activation related pseudoallergy
CDC: Center of Disease Control
DSPC: Phospholipid distearoylphosphatidylcholine
DtaP: Diphtheria–tetanus–pertussis
Flu: Influenza
HbsAg: Hepatitis B surface antigen
HPV: Human papillomavirus
MHRA: Medicines and Healthcare Products Regulatory Agency
MMR: Measles, mumps, rubella
MMRV: Measles, mumps, rubella, varicella
PEG: Polyethylene glycol
TBE: Tick-born encephalitis
Tdap: Tetanus-diphteria-pertussis
VAERS: Vaccine Adverse Event Reporting System

## References

[1] PharmaWiki Medikamente und Gesundheit. Zusatzstoffe. https://www.pharmawiki.ch/wiki/index.php?wiki=Zusatzstoffe. Accessed 4 Apr 2022.

[2] EDQM. European pharmacopoeia (Ph. Eur. 10.7). As of 1 April 2022.

[3] EMEA/CHMP/QWP/396951/2006. Guideline on excipients in the dossier for application for marketing authorisation of a medicinal product—Revision 2. Effective since 01.01.2008. https://www.ema.europa.eu/en/documents/scientific-guideline/guideline-excipients-dossier-application-marketing-authorisation-medicinal-product-revision-2_en.pdf. Accessed 4 Apr 2022.

[4] EU Guideline on Excipients in the labelling and package leaflet of medicinal products for human use. Effective since 3/2018. https://www.ema.europa.eu/en/human-regulatory/marketing-authorisation/product-information/reference-guidelines/excipients-labelling. Accessed 4 Apr 2022.

[5] EMA/CHMP/302620/2017 Rev. 1. Annex to the European Commission guideline on Excipients in the labelling and package leaflet of medicinal products for human use’ (SANTE-2017-11668). Effective since 11/2019. https://www.ema.europa.eu/en/documents/scientific-guideline/annex-european-commission-guideline-excipients-labelling-package-leaflet-medicinal-products-human_en.pdf. Accessed 4 Apr 2022.

[6] European Commission. A guideline on summary of product characteristics (SmPC), September 2009; included in: The rules governing medicinal products in the European Union Volume 2C notice to applicants. https://ec.europa.eu/health/system/files/2016-11/smpc_guideline_rev2_en_0.pdf. Accessed 3 Apr 2022.

[7] Radice A, Carli G, Macchia D, Farsi A (2019). Allergic reactions after vaccination: translating guidelines into clinical practice. Eur Ann Allergy Clin Immunol.

[8] Sampath V, Rabinowitz G, Shah M, Jain S, Diamant Z, Jesenak M (2021). Vaccines and allergic reactions: The past, the current COVID-19 pandemic, and future perspectives. Allergy.

[9] Weißer K, Barth I, Keller-Stanislawski B (2009). Sicherheit von Impfstoffen. Bundesgesundheitsblatt Gesundheitsforschung Gesundheitsschutz.

[10] Dreskin SC, Halsey NA, Kelso JM, Wood RA, Hummell DS, Edwards KM (2016). International Consensus (ICON): allergic reactions to vaccines. World Allergy Organ J.

[11] Oberle D, Pavel J, Rieck T, Weichert S, Schroten H, Keller-Stanislawski B (2016). Anaphylaxis after immunization of children and adolescents in Germany. Pediatric Infect Dis J.

[12] Shimabukuro T, Cole M, Su JR (2021). Reports of anaphylaxis after receipt of mRNA COVID 19 Vaccines in the US—December 14, 2020–January 18. JAMA.

[13] PEI. Sicherheitsbericht vom 07.02.2022. Verdachtsfälle von Nebenwirkungen und Impfkomplikationen nach Impfung zum Schutz vor COVID-19 seit Beginn der Impfkampagne am 27.12.2020 bis zum 31.12.2021. https://www.pei.de/SharedDocs/Downloads/DE/newsroom/dossiers/sicherheitsberichte/sicherheitsbericht-27-12-20-bis-31-12-21.pdf. Accessed 4 Apr 2022.

[14] CDC. Allergic Reactions Including Anaphylaxis After Receipt of the First Dose of Pfizer-BioNTech COVID-19 Vaccine—United States. December 14–23, 2020. https://www.cdc.gov/mmwr/volumes/70/wr/mm7002e1.htm. Accessed 4 Apr 2022.

[15] Rüggeberg JU, Gold MS, Bayas JM, Blum MD, Bonhoeffer J, Friedlander S (2007). Anaphylaxis: case definition and guidelines for data collection, analysis, and presentation of immunization safety data. Vaccine.

[16] Novadzki IM, Rosario Filho N (2010). Anaphylaxis associated with the vaccine against measles, mumps and rubella. Rev Saude Publica.

[17] RKI-Homepage. Impfungen bei Vorerkrankungen: Häufig gestellte Fragen und Antworten. Kann bei bestehender Hühnereiweißallergie geimpft werden? as of: 14.12.2012. https://www.rki.de/SharedDocs/FAQ/Impfen/AllgFr_Grunderkrankungen/FAQ-Liste_Impfen_und_Grunderkrankungen.html#FAQId2407616. Accessed 2 Apr 2022.

[18] Kelso JM (2014). Administering influenza vaccine to egg-allergic persons. Expert Rev Vaccines.

[19] Turner PJ, Southern J, Andrews NJ, Miller E, Erlewyn-Lajeunesse M (2015). Safety of live attenuated influenza vaccine in young people with egg allergy: multicentre prospective cohort study. BMJ.

[20] Worm M, Reese I, Ballmer-Weber B, Beyer K, Bischoff SC, Bohle B, Brockow K (2021). Update of the S2k guideline on the management of IgE-mediated food allergies. Allergol Sel.

[21] RKI-Homepage. Grippeschutzimpfung. Was ist bei der Influenza-Impfung von Personen mit einer Hühnereiweiß-Allergie zu beachten? As of: 07.09.2021. https://www.rki.de/SharedDocs/FAQ/Impfen/Influenza/faq_ges.html;jsessionid=159EFB3CF6636D740C28A77A078997E8.internet062#FAQId6948464. Accessed 2 Apr 2022.

[22] Centers for Disease Control and Prevention. Flu vaccine and people with egg allergies. https://www.cdc.gov/flu/prevent/egg-allergies.htm#recommendations. Accessed 2 Apr 2022.

[23] Kuritzky LA, Pratt M (2015). Systemic allergic contact dermatitis after formaldehyde-containing influenza vaccination. J Cutan Med Surg.

[24] de Silva R, Dasanayake WMDK, Wickramasinhe GD, Karunatilake C, Weerasinghe N, Gunasekera P (2017). Sensitization to bovine serum albumin as a possible cause of allergic reactions to vaccines. Vaccine.

[25] Pagán JA, Postigo I, Rodríguez-Pacheco JR, Peña M, Guisantes JA, Martínez J (2008). Bovine serum albumin contained in culture medium used in artificial insemination is an important anaphylaxis risk factor. Fertil Steril.

[26] Orta M, Ordoqui E, Aranzábal A, Fernández C, Bartolomé B, Sanz ML (2003). Anaphylactic reaction after artificial insemination. Ann Allergy Asthma Immunol.

[27] Mackensen A, Dräger R, Schlesier M, Mertelsmann R, Lindemann A (2000). Presence of IgE antibodies to bovine serum albumin in a patient developing anaphylaxis after vaccination with human peptide-pulsed dendritic cells. Cancer Immunol Immunother.

[28] Kattan JD, Konstantinou GN, Cox AL, Nowak-Węgrzyn A, Gimenez G, Sampson HA (2011). Anaphylaxis to diphtheria, tetanus, and pertussis vaccines among children with cow’s milk allergy. J Allergy Clin Immunol.

[29] Slater JE, Rabin RL, Martin D (2011). Comments on cow’s milk allergy and diphtheria, tetanus, and pertussis vaccines. J Allergy Clin Immunol.

[30] Nilsson L, Brockow K, Alm J, Cardona V, Caubet JC, Gomes E (2017). Vaccination and allergy: EAACI position paper, practical aspects. Pediatr Allergy Immunol.

[31] DiMiceli L, Pool V, Kelso JM, Shadomy SV, Iskander J (2006). Team VAERS. Vaccination of yeast sensitive individuals: review of safety data in the US vaccine adverse event reporting system (VAERS). Vaccine.

[32] Brotherton JML, Gold MS, Kemp AS, McIntyre PB, Burgess MA, Campbell-Lloyd S (2008). Anaphylaxis following quadrivalent human papillomavirus vaccination. Can Med Assoc J.

[33] Zhou ZH, Stone CA, Jakubovic B, Phillips EJ, Sussman G, Park J (2021). Anti-PEG IgE in anaphylaxis associated with polyethylene glycol. J Allergy Clin Immunol Pract.

[34] Kozma GT, Shimizu T, Ishida T, Szebeni J (2020). Anti-PEG antibodies: Properties, formation, testing and role in adverse immune reactions to PEGylated nano-biopharmaceuticals. Adv Drug Deliv Rev.

[35] Cabanillas B, Akdis C, Novak N (2021). Allergic reactions to the first COVID-19 vaccine: a potential role of polyethylene glycol?. Allergy.

[36] Krantz MS, Liu Y, Phillips EJ, Stone CA (2021). COVID-19 vaccine anaphylaxis: PEG or not?. Allergy.

[37] Stone CA, Liu Y, Relling MV, Krantz MS, Pratt AL, Abreo A (2019). Immediate hypersensitivity to polyethylene glycols and polysorbates: more common than we have recognized. J Allergy Clin Immunol Pract.

[38] Cabanillas B, Akdis C, Novak N (2021). COVID-19 vaccine anaphylaxis: IgE, complement or what else? A reply to: COVID-19 2 vaccine anaphylaxis: PEG or not?. Allergy.

[39] Wenande E, Garvey LH (2016). Immediate-type hypersensitivity to polyethylene glycols: a review. Clin Exp Allergy.

[40] Del Moral MG, Martínez-Naves E (2017). The role of lipids in development of allergic responses. Immune Netw.

[41] Lukawska J, Mandaliya D, Chan AWE, Foggitt A, Bidder T, Harvey J (2019). Anaphylaxis to trometamol excipient in gadolinium-based contrast agents for clinical imaging. J Allergy Clin Immunol Pract.

[42] Szebeni J (2014). Complement activation-related pseudoallergy: a stress reaction in blood triggered by nanomedicines and biologicals. Mol Immunol.

[43] Inglut CT, Sorrin AJ, Kuruppu T, Vig S, Cicalo J, Ahmad H (2020). Immunological and toxicological considerations for the design of liposomes. Nanomaterials (Basel).

[44] Mohamed M, Abu Lila AS, Shimizu T, Alaaeldin E, Hussein A, Sarhan HA (2019). PEGylated liposomes: immunological responses. Sci Technol Adv Mater.

[45] Hagemann PM, Nsiah-Dosu S, Hundt JE, Hartmann K, Orinska Z (2019). Modulation of mast cell reactivity by lipids: the neglected side of allergic diseases. Front Immunol.

[46] Coors EA, Seybold H, Merk HF, Mahler V (2005). Polysorbate 80 in medical products and nonimmunologic anaphylactoid reactions. Ann Allergy Asthma Immunol.

[47] Sellaturay P, Nasser S, Islam S, Gurugama P, Ewan PW (2021). PEG is a cause of anaphylaxis to the Pfizer/BioNTech mRNA COVID-19 vaccine. Clin Exp Allergy.

[48] Krantz MS, Kwah JH, Stone CA, Phillips EJ, Ortega G, Banerji A (2021). Safety evaluation of the second dose of mRNA COVID-19 vaccines in patients with immediate reaction the first dose. JAMA Intern Med.

[49] Warren CM, Snow TT, Lee AS, Shah MM, Heider A, Blomkalns A, Betts B (2021). Assessment of allergic and anaphylactic reactions to mRNA COVID-19 vaccines with confirmatory testing in a US regional health system. JAMA Netw Open.

[50] Medicines and Healthcare products Regulatory Agency (MHRA). Confirmation of guidance to vaccination centres on managing allergic reactions following COVID-19 vaccination with the Pfizer/BioNTech vaccine. Released: 09.12.2020. https://www.gov.uk/government/news/confirmation-of-guidance-to-vaccination-centres-on-managing-allergic-reactions-following-covid-19-vaccination-with-the-pfizer-biontech-vaccine. Accessed 8 Apr 2022.

[51] Weißer K, Kling K, Huth C, Keller-Stanislawski B, Mahler V (2021). Was ist bei positiver Allergieanamnese vor einer Impfung gegen COVID-19 zu beachten?. Bull Arzneimittelsicherh.

[52] PEI. Sicherheitsbericht vom 23.03.2021. Verdachtsfälle von Nebenwirkungen und Impfkomplikationen nach Impfung zum Schutz vor COVID-19. https://www.pei.de/SharedDocs/Downloads/DE/newsroom/dossiers/sicherheitsberichte/sicherheitsbericht-27-12-bis-12-03-21.pdf, Verdachtsfälle von Nebenwirkungen und Impfkomplikationen nach Impfung zum Schutz vor COVID-19 seit Beginn der Impfkampagne am 27.12.2020 bis zum 12.03.2021. Accessed 4 Apr 2022.

